# German Barcode of Life reveals unexpected diversity of Ceraphronoidea (Hymenoptera)

**DOI:** 10.3897/BDJ.13.e159561

**Published:** 2025-08-15

**Authors:** Marina Moser, Cristina Vasilița, Maura Haas-Renninger, Ecaterina Pirvu, Michael Haas, Lars Krogmann

**Affiliations:** 1 State Museum of Natural History Stuttgart, Stuttgart, Germany State Museum of Natural History Stuttgart Stuttgart Germany; 2 Biological Systematics (190w), Institute of Biology, University of Hohenheim, Stuttgart, Germany Biological Systematics (190w), Institute of Biology, University of Hohenheim Stuttgart Germany; 3 Center of Excellence for Biodiversity and Integrative Taxonomy (KomBioTa), Stuttgart, Germany Center of Excellence for Biodiversity and Integrative Taxonomy (KomBioTa) Stuttgart Germany; 4 Karlsruhe Institute of Technology, Eggenstein-Leopoldshafen, Germany Karlsruhe Institute of Technology Eggenstein-Leopoldshafen Germany; 5 Alexandru Ioan Cuza University, Iași, Romania Alexandru Ioan Cuza University Iași Romania

**Keywords:** dark taxa, biodiversity, Hill numbers, species richness estimators, Germany, GBOL

## Abstract

Insect populations still experience marked declines globally, contributing to the ongoing biodiversity crisis. Counteracting these declines requires sound taxonomic and ecological knowledge on all levels of biodiversity, from genes to species to ecosystems. The superfamily Ceraphronoidea (Hymenoptera) has remained relatively obscure due to complex challenges in exploring its diversity and ecological roles. Despite their ecological importance as parasitoids or hyperparasitoids, these wasps are under-represented in scientific exploration and conservation. In a case study within the German Barcode of Life (GBOL) Dark Taxa project, we aim to bridge this knowledge gap through a comprehensive taxonomic investigation covering 2,136 specimens of Ceraphronoidea across 18 locations in the putatively well-studied State of Baden-Württemberg (south-western Germany). Our study identifies a surprising species richness of at least 193 conjectural species, based on COI-barcoding clusters, extrapolates key species richness estimators for the German ceraphronoid fauna and records a species new to the German fauna: *Creatorspissicornis* (Hellén, 1966). By setting a foundational benchmark for Ceraphronoidea biodiversity, our research advocates for the inclusion of dark taxa in broader insect biodiversity assessments, contributing meaningfully to the discourse on conservation priorities and strategies.

## Introduction

Biodiversity is experiencing exceptionally rapid losses with current extinction rates at least 100 times higher than the background extinction rate (Pimm et al. 2006, Ceballos et al. 2015, Cowie et al. 2022). The extent to which insects have been affected was publicised in the “Krefeld study” in 2017, which reported a decline of 76% in the biomass of flying insects in protected areas throughout Germany over the course of 27 years (Hallmann et al. 2017). For insects, the declines can be ascribed to a combination of four major drivers: (1) habitat loss and conversion for intensive land use; (2) pollution, particularly by pesticides and fertilisers; (3) biological factors such as pathogens and introduced species; and (4) climate change (Sánchez-Bayo and Wyckhuys 2019). The knowledge necessary for understanding and counteracting these drivers to conserve insect biodiversity comes from taxonomic and ecological research. Researchers still have to describe, explore and understand the majority of biodiversity and its dynamics, from genetics to species and the ecosystem level (Kim and Byrne 2006, Shaw et al. 2025).

Insects make up over half of all described species and provide ecosystem services in all four categories, i.e. provisioning, regulating, cultural and supporting services (Ceballos et al. 2015), which makes them critical for ecosystem stability and human survival (Kim and Byrne 2006). At the same time, the majority of insect species are regularly neglected in conservation studies and policies (Shaw and Hochberg 2001, Ceballos et al. 2015, Habel et al. 2019). Amongst insects, parasitoid wasps are particularly species-rich with 115,000 described and up to 630,000 estimated species worldwide (Heraty 2017). Their parasitoid lifestyle is characterised by larval stages feeding on a host organism and eventually killing it to complete their development. Parasitoidism makes these wasps a key component of resilient ecosystems and balanced biological control programmes on account of their ability to effectively regulate the populations of their hosts (Godfray 1994).

Despite their ecological and economic importance, the taxonomy and biology of parasitoid wasps remain insufficiently understood. Very little is known about the life histories of most parasitoid Hymenoptera, including host ranges (Whitfield 1998, Moser et al. 2024), habitat requirements (Shaw 2006), phenology (Haas-Renninger et al. 2024), distribution patterns (Quicke 2012) and evolutionary history (Peters et al. 2017, Blaimer et al. 2023). In addition to their extraordinary species diversity, parasitoid wasps have been particularly hard to study using traditional morphological approaches. Many groups contain a plethora of morphologically highly similar taxa, which genetic markers recognise as cryptic species complexes rather than individual species (e.g. Smith et al. (2008), Chesters et al. (2012), Derocles et al. (2015), Gebiola et al. (2016)). At the other end of the spectrum, the significant morphological disparity of males and conspecific females has made their association, based on morphology alone, extremely difficult (Gokhman 2018).

Molecular methods add a significant source of information to integrative taxonomic research. DNA barcoding has become a standard method in characterising and identifying animal species and has significantly furthered taxonomy (Hebert et al. 2003). Recent technical (e.g. Srivathsan et al. (2021)) and conceptual (e.g. Hartop et al. (2022)) optimisations of this method facilitate the barcoding of species-rich samples, thus forming the basis for large-scale biodiversity research (Meier et al. 2025). Additionally, this method is particularly beneficial in dark taxa, i.e. taxa in which the majority of species richness is still undescribed and taxonomic expertise and resources are highly insufficient (Page 2016, Meier et al. 2025).

In the current study, we focus on the diversity of Ceraphronoidea (Hymenoptera), a vastly understudied superfamily of parasitoid wasps that serves as a case study for a dark taxon in the putatively well-studied State of Baden-Württemberg in south-western Germany. Globally, Ceraphronoidea are widespread and amongst the most commonly collected microhymenoptera (Masner 1993, Schmitt 2004, Haas-Renninger et al. 2024). With about 740 described species, the superfamily is moderately species-rich, though recent estimates suggest that the true species richness considerably exceeds this number (Salden and Peters 2023). As parasitoids and hyperparasitoids, ceraphronoids exhibit a broad host spectrum spanning nine insect orders (Moser et al. 2024). However, for over 80% of species, host information remains unknown, reflecting significant gaps in our understanding of their biology (Moser et al. 2024).

Most ceraphronoid wasps are minute with a body length ranging from 0.5 to 4 millimetres and their taxonomic study is further complicated by morphological challenges: their external morphology is relatively monotonous and lacks distinctive characters (Mikó et al. 2013, Moser et al. 2023). Additionally, the few potentially informative morphological traits available, such as body size and surface sculpture, are often affected by allometry (Mikó et al. 2013). Over the past decades, the dissection and examination of male genitalia have been considered the only reliable method for species identification and diagnosis (Mikó et al. 2013, Ulmer et al. 2018, Salden and Peters 2023). However, this approach is inherently limited by its applicability only to males, making females largely undiagnosable using morphological characters. Genetic barcoding represents a promising solution to overcome these limitations, yet an effective protocol for Ceraphronoidea has only recently been developed, with the introduction of customised primers specific to this taxon (Vasilița et al. 2022). Furthermore, the superfamily lacks specialists with in-depth knowledge of their taxonomy and biology. Notably, almost half of all species descriptions have been authored by only two taxonomists, Jean-Jacques Kieffer (1857–1925) and Paul Dessart (1931–2001) (Johnson and Musetti 2004). This shortage of expertise has led to a lack of well-curated and accessible identification resources (Masner 1993, Moser and Krogmann In press). Altogether, these challenges have fostered considerable taxonomic confusion and underscore why Ceraphronoidea exemplifies the key characteristics of a dark taxon, with the majority of species undescribed and fundamental aspects of their taxonomy, ecology and systematics poorly understood (Hartop et al. 2022, Moser et al. 2024).

Currently, the known species richness of Ceraphronoidea in Germany comprises 36 species: 12 species of Ceraphronidae and 24 species of Megaspilidae (Dessart 2001, Ulmer et al. 2018, Moser et al. 2023). To approximate a more realistic representation of the total species number, we present a comprehensive dataset of 2,136 barcode sequences of the superfamily Ceraphronoidea from Baden-Württemberg. Our primary objectives are: (1) to estimate species richness using molecular operational taxonomic units (mOTUs) as a proxy; (2) to add new records to the German ceraphronoid fauna; (3) to calculate ecological key parameters that help illuminate the biodiversity of the superfamily Ceraphronoidea and (4) to make the COI-barcodes publicly available as a basis for further research in the taxonomy, ecology, evolutionary biology and conservation of this particularly dark taxon. By making these data available and analysing key taxonomic and ecological parameters, we aim to promote the consideration of parasitoid wasps in broader applications, such as biodiversity monitoring, phylogenetics and biological control, aligning with global initiatives to document and conserve the Planet’s biodiversity.

## Material and methods

### Species sampling, barcoding and imaging

**Figure 1.**
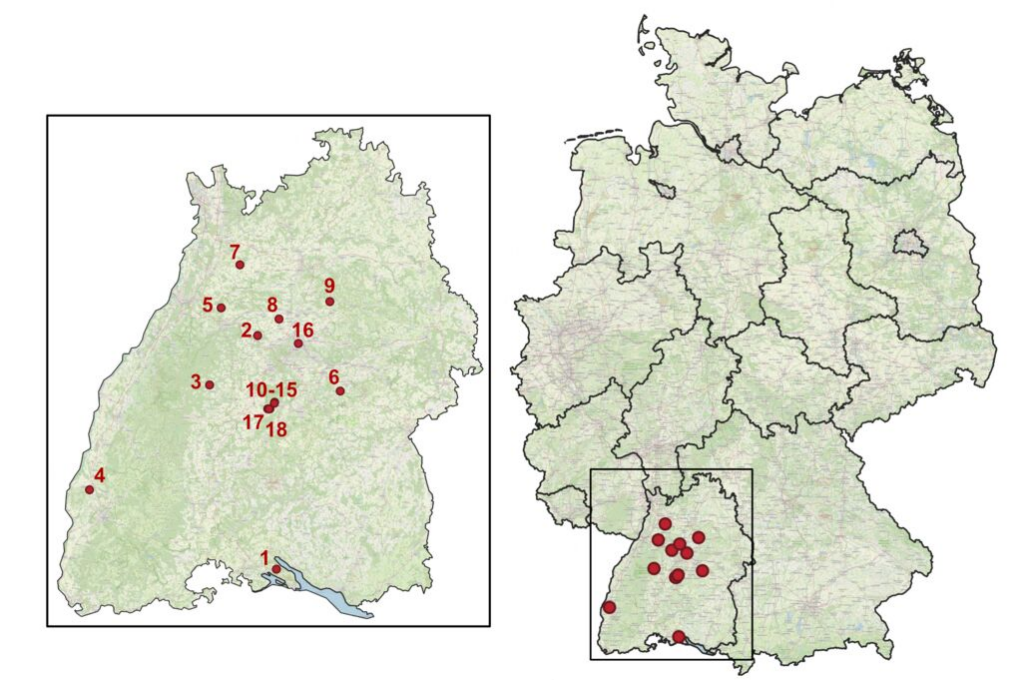
Map of Germany displaying the geographic distribution of sampling sites across the country. The highlighted region indicates the State of Baden-Württemberg, with markers indicating precise locations of sampling sites.

We generated barcode sequences of the mitochondrial cytochrome c oxidase subunit I (COI) for a total of 2,136 specimens through the megabarcoding approach (Wang et al. 2018). The specimens were collected across 18 localities in the State of Baden-Württemberg, encompassing the majority of its natural regions and ranging in elevation from 181 to 514 m a.s.l. (Fig. 1). The samples were collected between 2013 and 2022 and stored in 99.6% ethanol at the State Museum of Natural History Stuttgart (SMNS), Germany. Sampling was conducted with Malaise traps between the months of March and November and the samples were collected in 2-week intervals.

COI-barcoding was performed through HotSHOT DNA extractions as detailed in Vasilița et al. (In press) or by non-destructive extraction using the Qiagen DNeasy Blood and Tissue Kit, following the manufacturer’s protocol with minor changes as in Cruaud et al. (2019). Amplicons were then sequenced either by conventional Sanger sequencing or express barcoding and megabarcoding via MinION sequencing following the protocol of Vasilița et al. (2024) with a customised forward primer specific to Ceraphronoidea (Vasilița et al. 2022). Basecalling and demultiplexing followed the workflow provided by Srivathsan et al. (2021). Raw reads obtained by Sanger sequencing were assembled, trimmed and proofread with Geneious Prime. Sequences were aligned in Mega12 (Kumar et al. 2024) using the ClustalW method. The same software was used to calculate mean and pairwise genetic distances. DNA barcodes and associated metadata have been released to BOLD (DOI: dx.doi.org/10.5883/DS-CERBW). Identifications of the species recorded for the first time in Germany presented herein are based on results of the identification engine of BOLD V4 (https://v4.boldsystems.org/index.php/IDS_OpenIdEngine, last accessed: 02-03-2025) through comparison with published species-level barcode records and morphological identification.

Specimens were imaged using an MZ 16 APO Leica R microscope with an attached DXM 1200 Leica R camera and subsequent stacking of images in Helicon Focus (version 7.6.1; Helicon Soft Ltd, Kharkov, Ukraine).

### Species delimitation

Species delimitation was performed using three complementary algorithms to estimate the species diversity of Ceraphronoidea in Baden-Württemberg, based on mOTUs of the 2,136 COI-barcode sequences: first, ASAP (Assemble Species by Automatic Partitioning), which builds species partitions from single locus alignments using pairwise genetic distances (Puillandre et al. 2020). For further analyses, we selected the partitioning with the lowest ASAP score. This score incorporates both the probability of the partitioning and the width of the barcode gap, with lower scores indicating a better fit to the data. Second, ABGD (Automatic Barcode Gap Discovery), which builds species partitions from pairwise genetic distances by detecting the barcode gap (Puillandre et al. 2011). Both were run through the Spart-Explorer platform (Miralles et al. 2021), selecting the Jukes-Cantor (JC69) substitution model. Third, we used objective clustering in SpeciesIdentifier (Meier et al. 2006), using a 2% and 3% threshold, which corresponds to widely used thresholds for delineating mOTUs in DNA barcoding studies (e.g. Ratnasingham and Hebert (2007), Ratnasingham and Hebert (2013), Chimeno et al. (2023)), particularly in Braconidae (Hymenoptera) (Smith et al. 2012, Fernández‐Flores et al. 2013, Fagan‐Jeffries et al. 2018). Therefore, reporting the results at both the 2% and 3% thresholds allows for direct comparison with previous studies. For further analyses, we selected the less conservative 3% threshold, which aligns with the higher end of this standard range.

### Diversity index calculation

Statistical analysis was performed using R (version 4.2.1) in RStudio (version 2023.06.1) (R Core Team 2023). The following packages were used: vegan (Oksanen et al. 2001), dplyr (Wickham et al. 2023), ggplot2 (Wickham 2009), reshape2 (Wickham 2007), iNEXT (Hsieh et al. 2022), SpadeR (Chao et al. 2016), SPECIES (Wang 2024), hilldiv (Alberdi and Gilbert 2019) and BiodiversityR (Kindt 2025).

Species richness and abundance indices were calculated, based on the mOTUs described above. Sampling coverage was assessed by extrapolating the number of undetected species through non-parametric richness indices, namely the Chao1 estimator (Chao 1984), which takes into consideration all abundances down to singletons, and ACE (Abundance-based coverage; Chao and Lee (1992)), which divides the dataset into abundant taxonomic units (i.e. those which occur more than 10 times) and rare taxonomic units. Species accumulation curves were calculated for the dataset, assigning consecutive location IDs to each geographic location (Fig. 1), thus clustering multiple collection events from the same location. Diversity profiles were analysed using Hill numbers, i.e. species richness (q = 0), Shannon diversity (q = 1) and Simpson diversity (q = 2) (Hill 1973, Chao et al. 2014), ensuring a comprehensive assessment of biodiversity while addressing potential biases due to incomplete sampling.

To assess patterns in species composition across sampled locations, we performed non-metric multidimensional scaling (NMDS, Suppl. material 2) using the Bray-Curtis dissimilarity index (Ricotta and Podani 2017) to quantify compositional differences amongst sites. NMDS ordination was calculated using the *vegan* package in R (Oksanen et al. 2001) with k = 2, restricting ordination to two dimensions and a maximum of 1000 iterations to ensure convergence on a stable calculation (Suppl. material 2). Stress values were examined to ensure the goodness of fit.

## Results

The cluster analysis using ASAP returned a total of 193 subsets (ASAP score: 6.0) with a p-value of 9.999900e-06 and a threshold distance of 0.000018. The next two most likely ASAP partitionings return 193 and 185 subsets, both with an ASAP score of 14.5 (Table 1). The ten most likely partitions range from 185 to 214 clusters (average: 198.4). The three most probable ABGD partitioning all returned 211 initial partitions with prior maximum divergences of intraspecific diversity of p = 0.059948, p = 0.035938 and p = 0.021544. In contrast, SpeciesIdentifier returned 291 clusters at a threshold of 2.0% and 259 clusters at 3.0% (Table 1).

### Diversity indices

**Figure 2.**
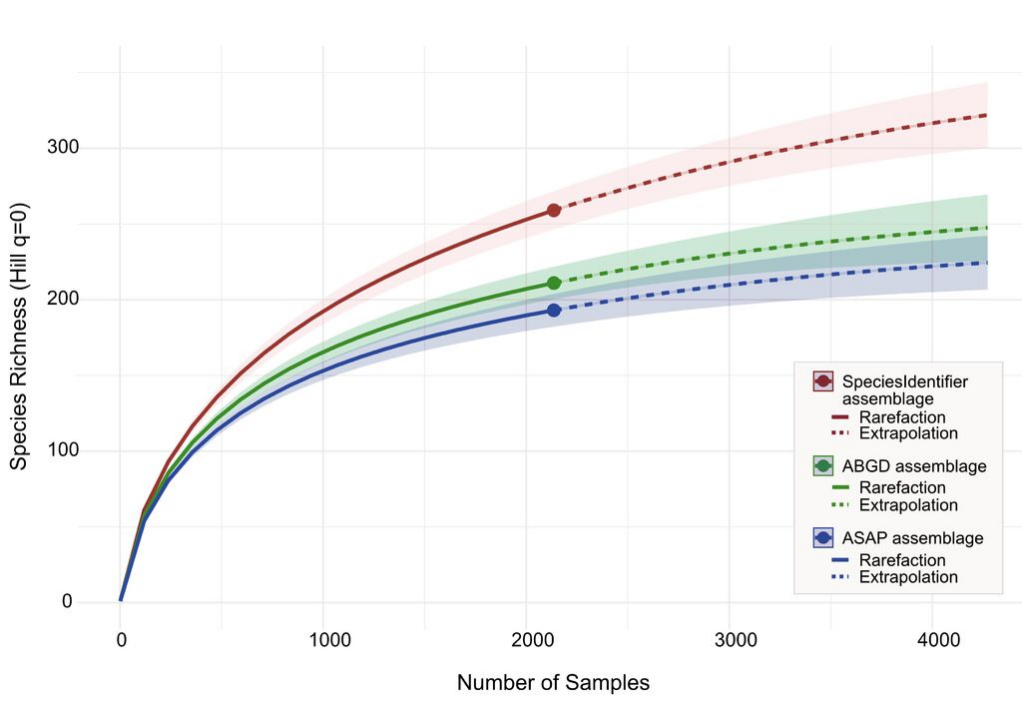
Rarefaction and extrapolation curve showing species richness as a function of sampling effort. The x-axis represents the number of samples, while the y-axis indicates the estimated species richness, based on clustering with SpeciesIdentifier (red), ABGD (green) and ASAP (blue). The solid portion of the curve represents observed richness (rarefaction), while the dashed portion represents projected richness (extrapolation).

Ecological analyses were conducted, based on the 193 ASAP, 211 ABGD and 259 SpeciesIdentifier clusters resulting from COI-barcodes of 2,136 individuals, which were treated as mOTUs (Fig. 2). The mean estimated sample coverage was 0.968 (ASAP: 0.976; ABGD: 0.972; SpeciesIdentifier: 0.956), indicating that the dataset captures the majority of species present in the Ceraphronoidea community of Baden-Württemberg. Species richness estimators suggested a higher true diversity, with Chao1 estimating a mean of 292.7 species (ASAP: 241.1; ABGD: 267.1; SpeciesIdentifier: 369.8) and ACE yielding a slightly lower extrapolation of a mean 284.9 species (ASAP: 234.9; ABGD: 259.9; SpeciesIdentifier: 360.0).

Taking into account both species richness and evenness, mean Shannon entropy estimates are 4.257 (Maximum Likelihood estimation, MLE) or 4.349 (Chao & Shen), corresponding to an effective number of species (i.e. Shannon diversity) between 71.475 (MLE) and 78.540 (Chao & Shen). Simpson’s diversity index results further supported high species evenness, with the inverse Simpson index estimated at 30.772 (minimum variance unbiased estimator, MVUE). The decrease in mean Hill numbers with increasing order, from 292.7 at q = 0 to 30.8 at q = 2, highlights the contribution of relatively few dominant species to overall community structure. These results provide a robust assessment of species diversity, accounting for both richness and evenness in the sampled population. Exact diversity indices are reported in Suppl. material 3.

### Taxonomic findings

The following species was identified morphologically and affirmed by comparison with identified sequences on BOLD and is a new record for the German fauna.

***Creatorspissicornis* (Hellén, 1966)**

*Creatorspissicornis* was originally described as *Lygocerusspissicornis* from Jomala and Nystad (Finland) by Hellén (1966). Since its original description, the species has undergone taxonomic re-assessment, resulting in a transfer to *Dendrocerus* (Dessart 1972) and later to its own monotypic genus, *Creator* (Alekseev 1980). The reclassification is based mainly on the shape of the syntergum, which is only slightly narrowed anteriorly, forming a broad collar and which is covered in strigose sculpture anteriorly (Alekseev 1980). Further notable characters are the coarse, mostly rugose sculpture as well as the relatively large body size (Alekseev 1980, Fergusson 1980).

Following its Finnish records, *C.spissicornis* has since been reported from additional European countries, including England, Sweden (Fergusson 1980), Belgium, France (Dessart 1972), Ukraine (Alekseev and Radchenko 2001), the Netherlands (observation by van Loon, M. (2023): https://waarneming.nl/observation/287666956/, accessed on 04-05-2025) and Norway (GBIF Secretariat 2023). The hosts of *C.spissicornis* are cyclorrhaphous Diptera (Moser et al. 2024).

The following three specimens were identified as *C.spissicornis*:

- Continent: Europe; country: Germany; countryCode: DE; stateProvince: Baden-Württemberg; locality: Lkr. Esslingen, Bissingen a. d. Teck, Eichhalde, Obstwiese; verbatimElevation: 513 m; decimalLatitude: 48.5070; decimalLongitude: 9.4926; samplingProtocol: Malaise trap; eventDate: 21/06/2014–10/08/2014; sex: female; lifeStage: adult; preparations: 99.6% ethanol; recordedBy: Rager, L.; identifiedBy: M. Moser; dateIdentified: 2025; accessionNumber: SMNS_Hym_Meg_000366.
- Continent: Europe; country: Germany; countryCode: DE; stateProvince: Baden-Württemberg; locality: Lkr. Karlsruhe, Östringen, NSG 2.217 Apfelberg; verbatimElevation: 181 m; decimalLatitude: 49.1675; decimalLongitude: 8.7903; samplingProtocol: Malaise trap; eventDate: 18/06/2019–02/07/2019; sex: female; lifeStage: adult; preparations: 99.6% ethanol; recordedBy: LUBW Insektenmonitoring; identifiedBy: M. Moser; dateIdentified: 2025; accessionNumber: SMNS_Hym_Meg_000565.
- Continent: Europe; country: Germany; countryCode: DE; stateProvince: Baden-Württemberg; locality: Lkr. Karlsruhe, Östringen, NSG 2.217 Apfelberg; verbatimElevation: 181 m; decimalLatitude: 49.1675; decimalLongitude: 8.7903; samplingProtocol: Malaise trap; eventDate: 30/07/2019–13/08/2019; sex: female; lifeStage: adult; preparations: 99.6% ethanol; recordedBy: LUBW Insektenmonitoring; identifiedBy: M. Moser; dateIdentified: 2025; accessionNumber: SMNS_Hym_Meg_000531.

**Figure 3.**
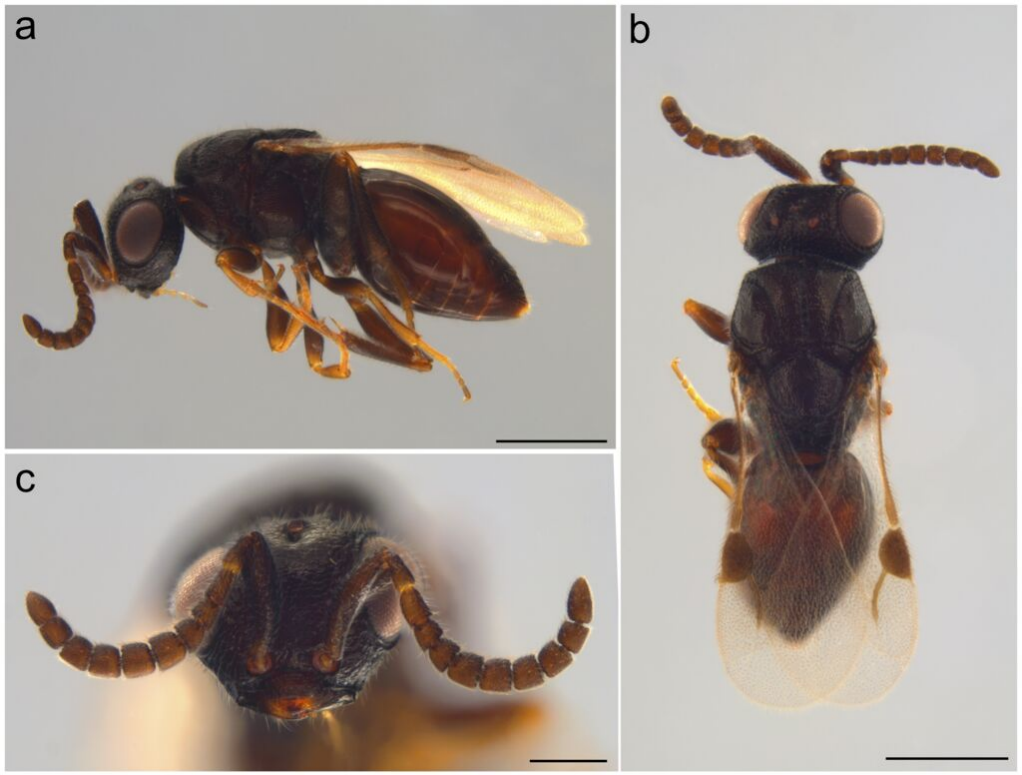
Female *Creatorspissicornis* (Hellén, 1966); SMNS_Hym_Meg_000531. **a** lateral habitus, scale bar: 500 μm. **b** Dorsal habitus, scale bar: 500 μm. **c** Antennae and head in frontal view, scale bar: 200 μm.

The similarity between the COI-sequences in our dataset and the closest reference sequences on BOLD (BIN ID: “BOLD:ADT2227”, sequence IDs: NOMEG358-21.COI-5P; NOMEG176-21.COI-5P) is 100% for both SMNS_Hym_Meg_000366 and SMNS_Hym_Meg_000531 (Fig. 3) and slightly lower at 99.83% for SMNS_Hym_Meg_000565. The overall mean distance between our COI-sequences and the BOLD sequences is 0.000653 with a maximum pairwise distance of 0.00167 between NOMEG176-21.COI-5P and SMNS_Hym_Meg_000565.

## Discussion

The parasitoid wasp superfamily Ceraphronoidea is a prime example of a dark taxon that has not received much scientific attention in the past. As a result, most species cannot be identified through genetic barcoding due to the missing connection between barcodes and formal scientific names in sequence reference databases, such as GenBank or BOLD (Page 2016). In Ceraphronoidea, which comprises about 740 described species worldwide, only 53 species (7.2%) are currently associated with barcodes deposited in BOLD (Megaspilidae: 47 species; Ceraphronidae: 6 species; https://v4.boldsystems.org/index.php/Taxbrowser_Taxonpage?taxid=125, accessed: 02-03-2025). The lack of reliably identified reference sequences often stems from a lack of taxonomic capacity, along with a general inaccessibility of older taxonomic literature (Page 2016), which is not retrievable online or not available in machine-readable format.

Like in many other dark taxa, species richness is much higher in Ceraphronoidea than the current number of described species would suggest. The results of the current study attest to at least 193 species of Ceraphronoidea in the State of Baden-Württemberg alone. This number is in stark contrast to the 36 species that are listed either in the most recent checklist of German Ceraphronoidea (Dessart 2001) or were recently described from Germany (Ulmer et al. 2018, Moser et al. 2023). This discrepancy could, in part, be attributed to the death of Paul Dessart, the only active Ceraphronoidea specialist worldwide at that time, in March 2001 (Pauly 2001). His death preceded the printing of the current checklist of German Hymenoptera in August 2001 (Dathe et al. 2001), for which he compiled the chapter on Ceraphronoidea (Dessart 2001).

For the State of Baden-Württemberg, our extrapolated rarefaction curves plateau at about 224 species (ASAP), 247 species (ABGD) and 322 species (SpeciesIdentifier) at twice the collection effort, i.e. 4,272 specimens (Fig. 2). According to our analysis, additional sampling would result in only little additional species detection beyond this point. These data, as well as the mean estimated sample coverage of 0.968, suggest that our sampling effort was sufficient to capture most of the community diversity. This is supported by the Chao1 estimator, which predicts a mean total of 292.7 species (ASAP: 241.1; ABGD: 267.1; SpeciesIdentifier: 369.8) species of Ceraphronoidea in Baden-Württemberg. However, it is important to acknowledge the limitations inherent to species richness analyses based on a single collection method. While Malaise traps are highly effective at sampling a broad spectrum of flying insect diversity and are well suited for investigating many parasitoid microhymenoptera (Darling and Packer 1988, Haas-Renninger et al. 2024), taxa that live mainly in leaf litter or canopy habitats could remain undetected. In particular, within the family Megaspilidae, some species of *Conostigmus* Dahlbom, 1858 and *Dendrocerus* Ratzeburg, 1852, as well as the females of *Lagynodes* Förster, 1841, are wingless and, therefore, unlikely to be captured effectively by Malaise traps. This suggests that further sampling with complementary methods such as pan traps, leaf litter extraction or targeted sweep netting would be required to capture the full species richness of Megaspilidae.

Notably, our mean Chao1 estimate of 292.7 species already exceeds the 259 described species of Ceraphronoidea known to occur in the whole Palaearctic (Johnson and Musetti 2004). However, this number is obviously an under-representation of the true species diversity of the Palaearctic: estimates for Europe have assumed about 400 species, cautioning that, for Ceraphronoidea, estimates are precarious at best due to the poor state of taxonomic knowledge (Ulrich 1999). As a model region diverse in habitats, geology and land-use types, Baden-Württemberg is estimated to be home to 75% of the Hymenoptera species found in Germany (Dathe et al. 2001, Dathe and Blank 2004). Based on the Chao1 estimations presented in the current study, which project that the number of Ceraphronoidea species in Baden-Württemerg lies somewhere between a minimum of 241.1 (following ASAP clustering) and a maximum of 369.8 (following SpeciesIdentifier clustering) species, we would expect a total of 321.5–493.1 species of Ceraphronoidea in Germany, provided that the overall ratio of all Hymenoptera (Dathe et al. 2001, Dathe and Blank 2004) is extrapolatable to Ceraphronoidea. Using the total number of 154,000 described species of Hymenoptera (Aguiar et al. 2013, Huber 2017) as a worldwide estimate and Germany being home to 9,625 (i.e. 6.25%) (Dathe and Blank 2004) of these species, the total number of Ceraphronoidea in the world could consequently be extrapolated to 5,143 – 7,889 species. This estimate seems conservative, especially compared to a recent estimate of 12,000 – 21,000 species, based on Afrotropical species (Salden and Peters 2023).

This discrepancy could, however, be informative in validating the hypothesis of Mikó et al. (2016), who raise the question of whether Ceraphronoidea show a reverse latitudinal diversity gradient in species richness or whether this phenomenon is merely the result of sampling bias. The comparison of 221 recorded and 292.7 expected species in the State of Baden-Württemberg (47° – 49° latitude) versus 88 recorded and 122 expected species in three regions of equatorial Africa (-0.3° – 3° latitude) (Salden and Peters 2023) gives evidence for a reverse latitudinal diversity gradient. This would align with recent findings of an “anomalous” latitudinal gradient in Ichneumonoidea, which peaks at 50° latitude (Castellanos‐Labarcena et al. 2024). However, to draw any definitive conclusions on global distribution patterns of Ceraphronoidea, more comprehensive sampling, especially across latitudes and with standardised methods, is required.

The key findings of the current study are the unrecognised species richness of Ceraphronoidea in Baden-Württemberg and the addition of a new species record to the German checklist. Furthermore, the analysis of the current dataset provides crucial insights into the ecology of Ceraphronoidea: species distribution throughout the community is moderately even, leading to a mean Shannon diversity (i.e. effective number of species) of 77.2 species across the three clustering methods and the community is dominated by 30.8 species as denoted by the inverse Simpson’s index (Suppl. materials 1, 3). These indicators of community structure emphasise the need to move beyond purely taxonomic approaches and integrate macroecological methodologies to uncover hidden patterns of biodiversity and distribution.

Despite their taxonomic and ecological significance, these findings most likely do not fully represent the whole diversity of Ceraphronoidea in Baden-Württemberg. Similar sampling across more habitat types and with complementary sampling methods could help address potential sampling biases, as well as put the ecological indicators presented herein into a larger context. This larger dataset would not only refine current diversity estimates, but also allow for the calculation of robust β-diversity metrics to assess differences in species richness and composition across sites. Further, continuous or periodic sampling would provide a more complete picture of community diversity over time and allow for analyses of how changes in land use and environmental factors influence biodiversity.

Taxonomically, an interesting next step would be the identification of as many of the mOTUs as possible. On the one hand, this approach could reveal a wealth of previously undescribed species, which require formal naming and description in order to be made available for databasing, further taxonomic and ecological research, as well as conservation strategies. On the other hand, this work would enable the assignment of existing species names to mOTUs, allowing for the linkage of ecological and taxonomic knowledge acquired through previous research. In combination with the results of the current study, this next step would improve our understanding of the distribution and abundance of ceraphronoid wasps throughout Germany, which is the basis for a comprehensive assessment of the extent to which they are threatened by the same drivers that cause overall insect declines (Shaw and Hochberg 2001, Haas-Renninger et al. 2024).

## Conclusions

Altogether, our case study demonstrates that even allegedly well-studied regions in Central Europe still require basic taxonomic research. Only a fraction of the mOTU clusters in the dataset were identifiable to species level through COI barcoding, which illustrates that future taxonomic work is urgently required.

The broader significance of this study lies in its implications for biodiversity conservation. Traditionally, conservation efforts have focused mainly on well-studied taxa with sufficient data available to enable assessment under the IUCN Red List Criteria. Within this framework, lesser-known dark taxa, including many groups of parasitoid wasps and nematoceran Diptera, are frequently neglected due to their taxonomic disarray and the consequent lack of robust data. Yet, parasitoid wasps, such as Ceraphronoidea, play a major role in ecosystems, as indicated by the combination of high species diversity and moderately even community structure found in the current study. Given their high trophic level in food webs as highly specialised (hyper-)parasitoids, future biodiversity management and conservation frameworks should integrate parasitoids like Ceraphronoidea, moving beyond the obsolete dogma of waiting for complete taxonomic resolution before implementing conservation measures.

The discovery of unrecognised species richness amongst ceraphronoid wasps in our case study, conducted in a region previously believed to be well-documented, underscores the necessity for more comprehensive conservation strategies. Moving forward, integrating taxonomic and ecological analyses will provide valuable insights into patterns of biodiversity and distribution, bridging the knowledge gaps that have historically excluded these taxa from targeted conservation measures. Since filling these knowledge gaps requires substantial time, resources and expertise, it would be pragmatic for conservation strategies to prioritise overall habitat and ecosystem preservation. Focusing on maintaining habitat quality would benefit and protect the whole spectrum of biodiversity, covering both known and yet-to-be-discovered elements of biodiversity, ultimately fostering a comprehensive approach to maintaining ecosystem resilience.

## Supplementary Material

F282F6FD-CBE3-5CE3-9EDC-0DA01AFB9AB5 10.3897/BDJ.13.e159561.suppl1 Supplementary material 1 Supplementary Figure 1 Data type Image Brief description Biodiversity indices of the Ceraphronoidea community of Baden-Württemberg using ASAP clustering (a,b), ABGD clustering (c,d) and SpeciesIdentifier clustering (e,f). Species accumulation boxplots (a,c,e) plot the number of sampling sites (x-axis) against the cumulative number of species observed (y-axis). Boxplots represent variability (95%-confidence intervals) in species richness at each sampling effort level. Diversity profiles (b,d,f) are based on Hill numbers for different orders of diversity: q = 0 (i.e. species richness), 1 (Shannon diversity), 2 (Simpson diversity) (Chao et al. 2014), illustrating how diversity estimates change with varying levels of sensitivity to species abundances. File: oo_1329811.png https://binary.pensoft.net/file/1329811 Moser, M; Vasilița, C; Haas-Renninger, M; Pirvu, E; Haas, M; Krogmann, L

1F4E0EAC-697B-5D30-9BF5-1721117AFBBC 10.3897/BDJ.13.e159561.suppl2 Supplementary material 2 Supplementary Figure 2 Data type Image Brief description Non-metric multidimensional scaling (NMDS) ordination of species composition across sampled sites, based on Bray-Curtis dissimilarity of ASAP clustering. Each point represents a sampling site and the distance between points reflects differences in community composition. The ordination was performed with a maximum of 1000 iterations in two dimensions (k = 2), achieving a final stress value of 0.088. Points are colour-coded by habitat type to illustrate differences in species composition across habitats. File: oo_1329812.png https://binary.pensoft.net/file/1329812 Moser, M; Vasilița, C; Haas-Renninger, M; Pirvu, E; Haas, M; Krogmann, L

886ED892-EA0C-54A9-B1B5-7A53B827FE42 10.3897/BDJ.13.e159561.suppl3 Supplementary material 3 Supplementary Table 1 Data type Table Brief description Comparative analysis of diversity indices for varying q values using Chao & Jost (2015) and empirical Maximum Likelihood estimators, along with 95% confidence intervals. File: oo_1379383.pdf https://binary.pensoft.net/file/1379383 Moser, M; Vasilița, C; Haas-Renninger, M; Pirvu, E; Haas, M; Krogmann, L

## Figures and Tables

**Table 1.** Summary of the number of clusters identified by different approaches and respective parameters or indicators used for species delimitation. The methods compared are ASAP (Assemble Species by Automatic Partitioning, Puillandre et al. (2020)) with resulting ASAP scores, ABGD (Automatic Barcode Gap Discovery, Puillandre et al. (2011)) with respective barcode gaps and SpeciesIdentifier (Meier et al. 2006) with a 2% and 3% a priori threshold.

**Species delimitation method**	**Number of clusters**	**Indices / Parameters**
ASAP	193	asap score: 6.0
ASAP	193	asap score: 14.5
ASAP	185	asap score: 14.5
ABGD	211	barcode gap distance: 0.059948
ABGD	211	barcode gap distance: 0.035938
ABGD	211	barcode gap distance: 0.021544
SpeciesIdentifier	291	threshold: 2.0%
SpeciesIdentifier	259	threshold: 3.0%
