# Supplementary figures and images for: German Barcode of Life reveals unexpected diversity of Ceraphronoidea (Hymenoptera)
Source: Biodivers Data J. 2025 Aug 15;13:e159561. doi: 10.3897/BDJ.13.e159561 (PMC12374168; doi:10.3897/BDJ.13.e159561)

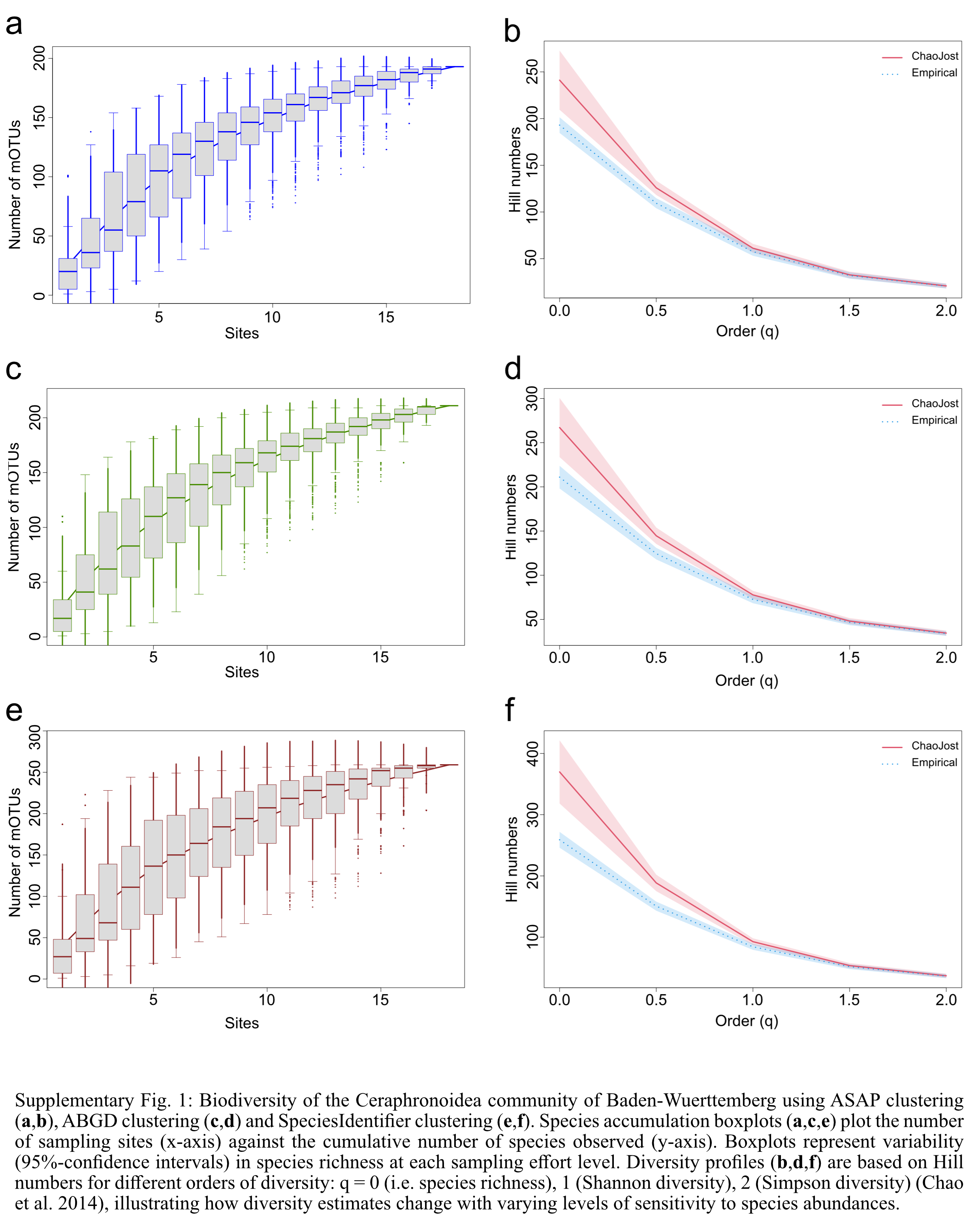

Supplement: Supplementary material 1 — Supplementary Figure 1 [file bdj-13-e159561-s001.png]

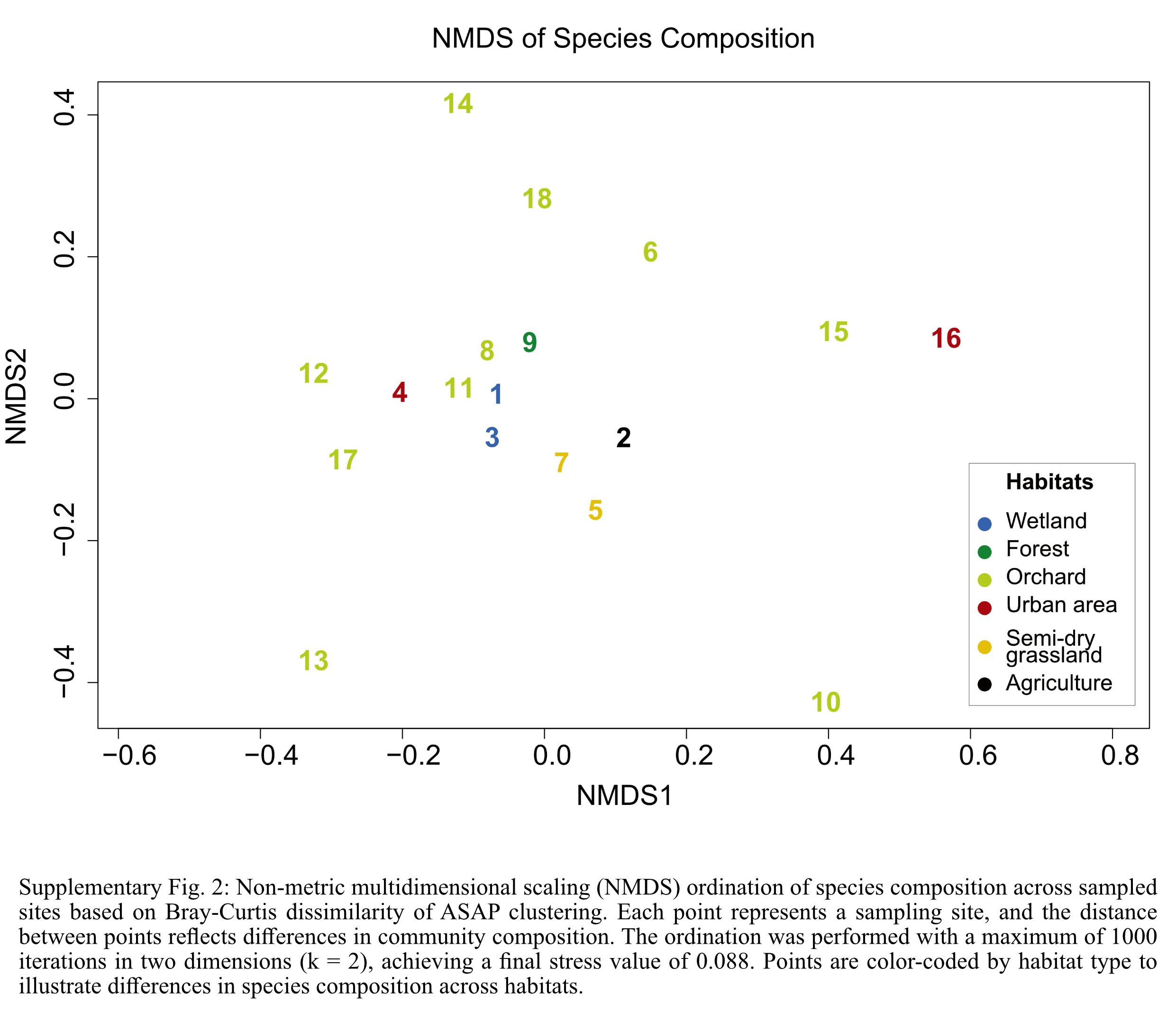

Supplement: Supplementary material 2 — Supplementary Figure 2 [file bdj-13-e159561-s002.png]
