# Supplementary material for: German Barcode of Life reveals unexpected diversity of Ceraphronoidea (Hymenoptera)
Source: Biodivers Data J. 2025 Aug 15;13:e159561. doi: 10.3897/BDJ.13.e159561 (PMC12374168; doi:10.3897/BDJ.13.e159561)
Supplement: Supplementary material 3 — Supplementary Table 1 [file bdj-13-e159561-s003.pdf]

Supplementary Table 1: Comparative analysis of diversity indices for varying q values using Chao & Jost (2015) and empirical maximum likelihood estimators, along with 95% confidence intervals.

**q**	**Clustering method**	**ChaoJost (Chao & Jost 2015)**	**Empirical (maximum likelihood estimator)**
0 (species richness)	ASAP	241.144 (95% CI: 209.447 – 272.841)	193
	ABGD	267.119 (95% CI: 236.367 – 297.871)	211
	SpeciesIdentifier	369.833 (95% CI: 324.022 – 415.644)	259
0.5 (effective number of species)	ASAP	125.869 (95% CI: 118.166 – 133.572)	109.363 (95% CI: 103.975 – 114.751)
	ABGD	144.656 (95% CI: 135.558 – 153.754)	124.444 (95% CI: 118.437 – 130.451)
	SpeciesIdentifier	188.552 (95% CI: 175.024 – 202.080)	150.599 (95% CI: 143.002 158.196)
1 (Shannon diversity)	ASAP	61.181 (95% CI: 56.499 – 65.863)	57.519 (95% CI: 53.109 – 61.929)
	ABGD	77.756 (95% CI: 72.646 – 82.866)	72.604 (95% CI: 67.982 – 77.226)
	SpeciesIdentifier	92.658 (95% CI: 87.176 – 98.140)	84.301 (95% CI: 79.419 – 89.183)
1.5 (Simpson diversity)	ASAP	32.679 (95% CI: 29.167 – 36.191)	31.966 (95% CI: 28.575 – 35.357)
	ABGD	48.108 (95% CI: 44.012 – 52.204)	46.744 (95% CI: 42.840 – 50.648)
	SpeciesIdentifier	53.576 (95% CI: 49.417 – 57.735)	51.762 (95% CI: 47.817 – 55.707)
2 (Inverse Simpson Diversity)	ASAP	20.834 (95% CI: 18.288 – 23.380)	20.642 (95% CI: 18.143 – 23.141)
	ABGD	34.554 (95% CI: 30.991 – 38.117)	34.020 (95% CI: 30.566 – 37.474)
	SpeciesIdentifier	36.929 (95% CI: 33.466 – 40.392)	36.318 (95% CI: 32.966 – 39.670)
